# Reconstruction of a maxillary defect with a fibula graft and titanium mesh using CAD/CAM techniques.

**DOI:** 10.1186/1746-160X-6-16

**Published:** 2010-07-19

**Authors:** Bernd Lethaus, Peter Kessler, Roland Boeckman, Lucas J Poort, Rene Tolba

**Affiliations:** 1 Department of Cranio-Maxillofacial Surgery, Maastricht University Medical Center, The Netherlands; 2 Institute for Laboratory Animal Science and Experimental Surgery, RWTH Aachen University, Aachen, Germany

## Abstract

We present a case of maxillary and orbital floor reconstruction with a microvascular fibula graft and an individualized titanium mesh. Both were planned virtually; templates were made by rapid prototyping. The postoperative computertomography scans showed that the planned positions were achieved correctly. This case report illustrates maxillary reconstruction performed with a special template technique and demonstrates the possibilities of computer aided design/computer aided manufacturing (CAD/CAM) applications in reconstructive surgery.

## Background

The use of virtual planning to restore tissue that was lost due to trauma or tumor surgery is becoming more popular in reconstructive surgery. Particularly in complex anatomical situations involving different sorts of tissue, the use of CAD/CAM applications facilitates planning and execution. This method is widespread in craniomaxillofacial surgery, but also other specialties are using this techniques in their clinical routine [1,2]. The rapid prototyping approach allows the creation of any desired three-dimensional design, which is created virtually using computer software. Models and templates built through rapid prototyping allow the surgeon to bring the planning to the operating theatre and close the gap between set-up and execution. Here, we report a case of reconstruction with a special technique for virtual planning and rapid prototyping. We also want to demonstrate the ability to plan and execute the restoration of an anatomically complex area with functional demands.

### Case presentation

**Figure 1:**
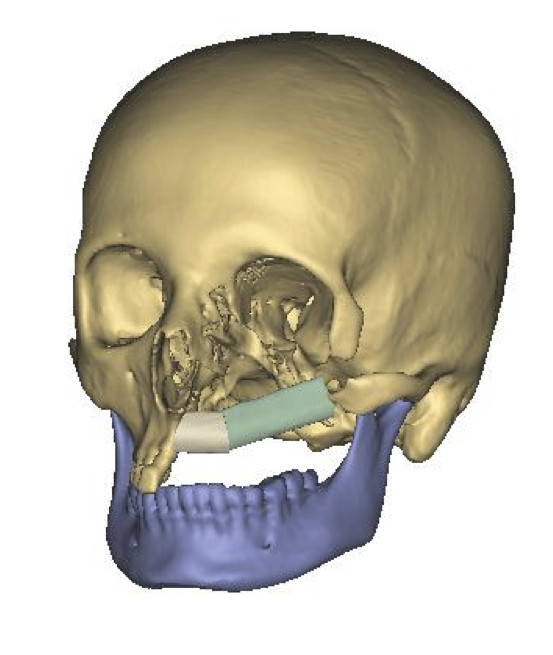
**Preoperative situation with prosthetic ideal bone position**.

**Figure 2:**
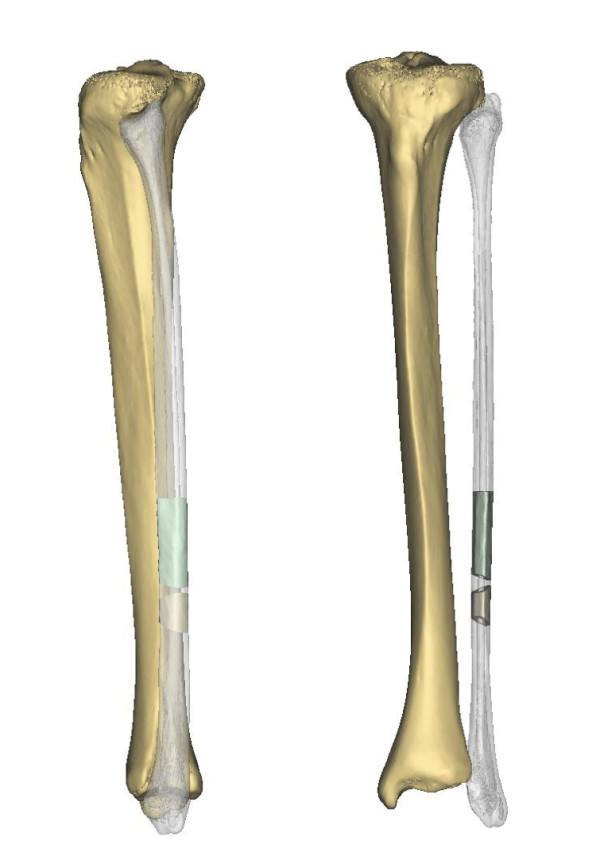
**Necessary bone needed to match with left fibula**.

**Figure 3:**
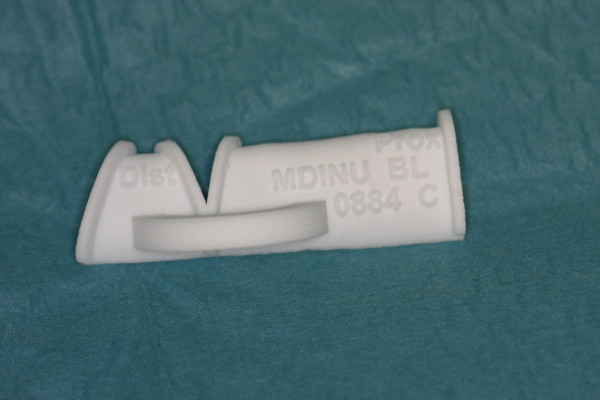
**Rapid prototyped template, which defined the graft length and angle of junction**.

**Figure 4:**
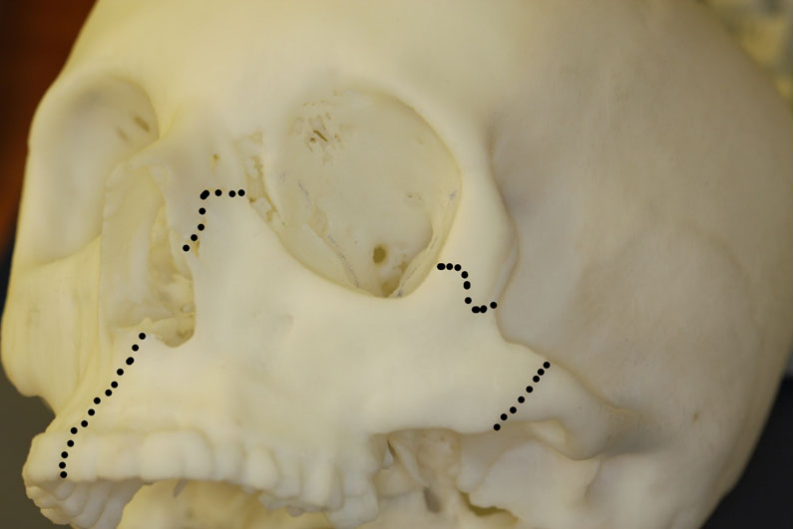
**Rapid prototyped skull with the mirrored intact zygomatic region inserted (marked with dots)**.

A 25-year-old female was introduced to our department seeking reconstruction of her left maxilla. At the age of 17, an ossifying cementoblastoma was diagnosed, and the patient underwent hemimaxillectomy. The orbital floor next to the maxilla had also been removed, which resulted in an enophthalmus and a collapsed cheek. The open connection between the nasal and oral cavities was treated with a removable prosthesis. The patient complained about the prosthesis size and its heaviness, which made chewing difficult and gave the speech a nasal tone. According to the patient, this was a massive reduction of her quality of life. To reduce the defect and to reconstruct the processus alveolaris, a microvascular fibula flap was selected for transfer. An individually premolded titanium mesh was used to reconstruct the floor of the eye. "Backward" planning was used to find the best position of the bony part. The position of the mandibula was predefined as the ideal position for the implants, which then predefined the ideal position for the transferred bone (Figure 1). A computertomography (CT) scan of both legs was performed, and the necessary bony shape was virtually matched with the patient's left fibula (Figure 2). To achieve the desired lengths and angles at the fibula's resection and split sites, a rapid prototyped template (figure 3) was manufactured by Materialize^© ^(Leuven, Belgium). To reconstruct the orbital floor, the intact site of the skull was mirrored, and the missing bony part was identified (figure 4). Both parts were produced with rapid prototyping by IDEE^© ^(Instrument Development Engineering & Evaluation, Maastricht University, Maastricht, The Netherlands). The complete skull was then used to premold a titanium mesh, which was sterilized before surgery.

**Figure 5:**
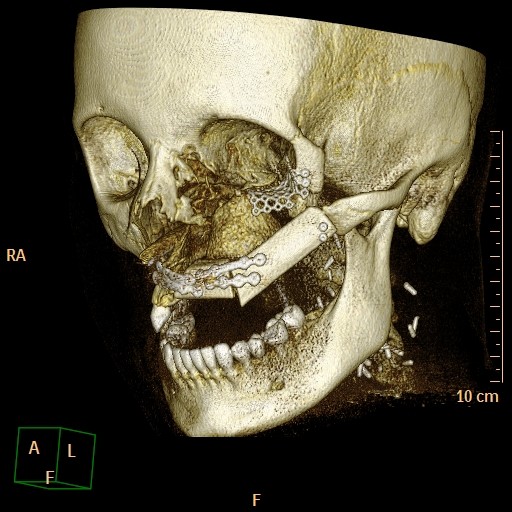
**Postoperativ CT 3D reconstruction**.

During the operation, the incision made previously was used to open the site. As planned, the fibula and the individualized titanium mesh were placed in the sites selected preoperatively. Both were fixed with osteosynthesis screws of 2.0 diameter (KLS Martin Tuttlingen, Germany). The fibula was reanastomized to the vena jugularis interna and the arteria carotis externa. Wound healing was uneventful for the following three weeks. A CT scan obtained two days postoperatively demonstrated the accuracy of the fibula insertion (figure 1, 5). The removal of the osteosynthesis material and the placement of dental implants will be performed six months after the operation.

## Discussion and Conclusion

This case demonstrates that CAD/CAM techniques can be of great value in planning and executing the reconstruction of resected or damaged tissue. The bone and the titanium mesh can be placed in the desired positions. Dental rehabilitation will take place after healing of the bony junctions is complete.

Two groups have recently demonstrated the efficacy of virtual planning and use of a rapid prototyped template to reconstruct the mandible with a fibula graft. These researchers presented favorable results concerning precision and outcome [3,4]. Compared to the mandible, the maxilla presents an even more complex area for reconstruction. Soft tissue covers most of the bony structures, especially the remaining bone at the skull base region, which is necessary for bone fixation. The anatomical proximity to vital structures further complicates the process of reconstruction.

We regard 3D models as a reasonable amendment in craniofacial reconstruction that offers multiple advantages. They facilitate surgical planning by demonstrating the anatomical characteristics of the tissue to be operated upon. By adding a haptic sensation, this approach optimizes preoperative planning. The surgeon achieves a better impression of the anatomical situation, the actual amount of bone and the demands on the reconstruction, which will result in a safer operation, shorter operation time and a more predictable result. We also use the models to explain and discuss the operation with our patients, providing them with a better understanding of the process and its possible outcomes.

Virtual planning and the use of rapid prototyping have been used mainly in craniomaxillofacial surgery. Because of the use of specialized software systems, application of this technique is limited to larger medical centers. The disadvantages are additional costs for software and computers and the additional time needed to plan the operation. Nevertheless, rapid prototyping is used in different areas of medicine. In the context of spine surgery, templates can be used to position cervical screws to ensure correct positioning that will avoid nerve damage [5,6]. In orthopedic surgery, templates can be used to navigate endoprostheses. Both hip and knee implants were positioned correctly after virtual planning by means of rapid prototyped templates [7,8]. Cardiosurgeons have described the benefits of using rapid prototyped models to visualize complex cardiac morphology or to build aortic stents for training [9-11]. Those examples should encourage more surgical specialties to use these techniques and to benefit from the advantages of preoperative planning.

## Consent

Written informed consent was obtained from the patient for publication of this case report and any accompanying images. A copy of the written consent is available for review by the Editor-in Chief of this journal.

## Competing interests

The authors declare that they have no competing interests.

## Authors' contributions

BL was responsible for a part of the operation and drafted the manuscript. LP was responsible for the planning and manufacturing of the templates and the titanium mesh. PK and RB were responsible for a part of the operation. RT conceived the report, participated in its coordination and helped to draft the manuscript. All authors read and approved the final manuscript.
